# Older adults’ demands for sports parks: a Kano model study based on sports parks in Shandong Province of China

**DOI:** 10.3389/fpubh.2025.1703037

**Published:** 2025-12-29

**Authors:** Rui Miao, Xin Yang, Dianbo Zhang

**Affiliations:** 1 School of Graduate Education, Shandong Sport University, Jinan, China; 2 School of Physical Education, Beijing Sport University, Beijing, China

**Keywords:** sports parks, older adults, Kano model, active aging, public sport service

## Abstract

**Aim:**

With the rapid progression of population aging in China, community sports parks are becoming vital venues for older adults’ physical activity and social interaction. However, deficiencies in age-friendly design remain evident. This study investigates older adults’ service demands in community sports parks using the Kano model, aiming to identify attributes that shape satisfaction and inform the development of older adults friendly facilities.

**Methods:**

A survey was conducted in three representative parks in Jinan, Rizhao, and Weihai (Shandong Province) between May and July 2025. A total of 900 questionnaires were distributed, with 838 valid responses collected (93.1%). The instrument covered six dimensions and 25 service items in functional/dysfunctional pairs. Reliability and validity tests were performed with SPSS 26.0, and Kano analysis was applied to classify demand attributes.

**Results:**

Indifferent attributes accounted for the largest proportion (36%), while attractive, must-be, and one-dimensional attributes represented 28, 20, and 16%, respectively. Public service facilities, sports facilities, multi-sharing, professional fitness guidance and sports skills training were identified as must-be requirements. Lighting system, environmental quality, maintenance management, greening facilities, social adaptability, health education, health monitoring activities were attractive attributes, while site protection, spatial comfort, recreational fitness facilities, recreational sports activities were one-dimensional expectations. Dimensional analysis highlighted that convenience and openness were critical to avoiding dissatisfaction, whereas environmental comfort was the strongest driver of satisfaction.

**Conclusion:**

Older adults’ needs for community sports parks are multi-layered, encompassing safety, accessibility, comfort, sociability, and activity diversity. Essential functions must be guaranteed, while improvements in comfort, enrichment of activities, and intergenerational integration are crucial to enhancing satisfaction and participation. These findings provide empirical evidence to guide the age-friendly transformation of community sports parks and support the broader implementation of active aging strategies.

## Introduction

According to projections from the Ministry of Civil Affairs, by the end of the “14th Five-Year Plan” period, the number of Chinese citizens aged 60 and above will reach 300 million, accounting for more than 20% of the total population, marking the transition to a moderately aging society (1). By 2040, this figure is expected to rise to 402 million, or 28% of the population (2). Against this demographic backdrop, the construction of age-friendly communities has become a national priority to promote active aging. Policy documents such as the Call for Applications for the National Demonstration Program of Age-friendly Communities explicitly state that by 2025, 5,000 demonstration communities will be established nationwide, and by 2035, universal coverage of age-friendly communities will be achieved (3). Nevertheless, the continuous growth of the older adults population and their increasingly diverse needs present complex developmental challenges to communities, the fundamental units of society. In response, multiple sectors are actively promoting the agenda of active aging. The physical and spatial living environment is a crucial factor shaping older adults’ sense of well-being; therefore, building age-friendly communities requires full consideration of residential settings, community infrastructure, and service provision.

Active aging should not only emphasize older adults’ physical and mental health and safety, but also highlight their proactive participation and contributions to society. Social participation represents the essence and core of active aging, enabling older adults to maintain dignity and continue realizing life’s value. Drawing on Maslow’s hierarchy of needs, belonging, social connection, and emotional support are key determinants of older adults’ social participation, forming the foundation for self-actualization. However, existing studies reveal that the level of social participation among older adults remains relatively low, particularly among those living alone. Thus, enhancing the social participation of older populations has become an urgent question of both academic interest and policy concern. Within this context, community sports parks as components of age-friendly communities provide new spaces for nationwide fitness and serve as important platforms to promote older adults’ social participation. Beyond meeting physiological and psychological needs, such parks offer diverse functional zones, safe exercise infrastructure, and human-centered ecological landscapes. Together, these features create spaces that encourage broad social interaction, enrich emotional experience, and enhance older adults’ participation in community life, thereby fostering interpersonal connections across society.

At present, scholarly research on age-friendly community construction has primarily focused on aspects such as park characteristics (4), exercise spaces (5), outdoor fitness equipment (6), and the role of sports parks in enhancing social interaction (7). However, relatively few studies have specifically addressed the needs of older adults within community sports parks. This study, situated in the context of active aging, seeks to clarify the contemporary significance of age-friendly sports parks, analyze their current status in Shandong Province, identify bottlenecks in their development, and outline practical pathways for improvement. The aim is to provide both theoretical grounding and practical reference for the construction of age-friendly community sports parks in China. As newly designated spaces for older adults’ fitness and recreation, age-friendly sports parks integrate the physiological and psychological characteristics of the older adults, take into account requirements for rehabilitation and health maintenance, and balance appropriate exercise intensity. By incorporating older adults friendly fitness elements and creating dedicated activity zones, these parks ensure the protection of older adults’ rights to fitness services while advancing the broader goal of building a harmonious society.

## Literature review

### Sport park

The construction of sports parks has become a new focal point for enhancing urban image and advancing sports development (8), Increasing attention has been given to the sports services provided by urban parks (9). Urban parks are widely recognized as important venues for physical activity among older urban residents (10). They not only offer opportunities for exercise and social interaction but also play a critical role in improving public health (11). The development of sports parks contributes to ecological improvements, meets the public’s evolving expectations regarding sports culture, enhances residents’ life satisfaction (12), and is associated with significant physical, psychological, and social health benefits (13). Studies have shown that participants report relatively high levels of satisfaction with sports parks. Factor analysis further indicates that sports facilities and maintenance management are the two most important factors influencing residents’ willingness to use such parks. These findings provide valuable guidance for future planning and construction of sports parks (14). Community sports parks also have a positive impact on residents’ subjective well-being. To increase multifunctionality, they should incorporate a diverse range of facilities (15). As vital community resources for public health, parks with more amenities and higher levels of accessibility are associated with greater satisfaction (16).

The lack of space for physical activity (PA) is one of the most significant non-behavioral barriers, contributing to a 10% shortfall in leisure-time physical activity among individuals aged 20 and above (17). As scarce yet essential resources in compact and vibrant urban areas, city parks must be planned and designed with an understanding of the needs, preferences, and aspirations of diverse visitors. Inclusive planning, design, and management of green spaces are key strategies to achieve this goal. Parks should also be integrated with surrounding areas to generate additional value for users (18). However, due to uneven population distribution, the allocation of sports parks is currently imbalanced; specifically, the number of existing parks in different districts does not align with local population sizes (19).

Community sports parks represent an important strategy for improving healthy community environments (20), as they are expected to meet the rising demand for activities essential to both residents’ well-being and urban development (21). Older adults, in particular, prefer using trails, paved open spaces (22), and natural areas for exercise. Modernizing sports parks to incorporate more green spaces can enhance their attractiveness to older users, encourage them to spend more time outdoors, and ultimately improve their health and well-being (23). Research shows that older adults devote approximately 62.43% of their park visits to physical exercise (24). Therefore, outdoor fitness facilities should be designed with priority given to older adults’ needs and preferences, encouraging regular participation in physical activity and potentially improving health and quality of life (25). Creating parks within walking distance of residential areas can further increase the frequency of physical activity among local residents (26).

The Kano model has been applied to analyze older adults’ demand for public sports services (27), to examine urban residents’ usage requirements for pocket parks (28), and to compare the effects of fitness facility service landscapes on consumer satisfaction in China and South Korea (29). Applying the Kano model to community sports parks provides a structured framework to better understand and address the diverse needs of older adults. By using Kano evaluation, service providers can identify attributes that most effectively enhance satisfaction and participation.

The Kano model categorizes product and service attributes into five types:

*Must-be attributes*: These represent the basic functions expected by users and are critical to service quality. When adequately provided, satisfaction does not necessarily increase, but their absence leads to significant dissatisfaction among older adults with respect to sports park services.

*One-dimensional attributes*: These directly correlate with satisfaction—fulfillment leads to satisfaction, while absence leads to dissatisfaction. For older adults, such attributes reflect expectations regarding the performance of sports park services, with higher levels of performance translating into higher satisfaction.

*Attractive attributes*: These features delight users when present but do not cause dissatisfaction when absent. Often unexpected, they can differentiate a service or facility from its competitors.

*Indifferent attributes*: Users remain neutral toward these attributes, as they do not substantially influence satisfaction or dissatisfaction.

**Figure 1:**
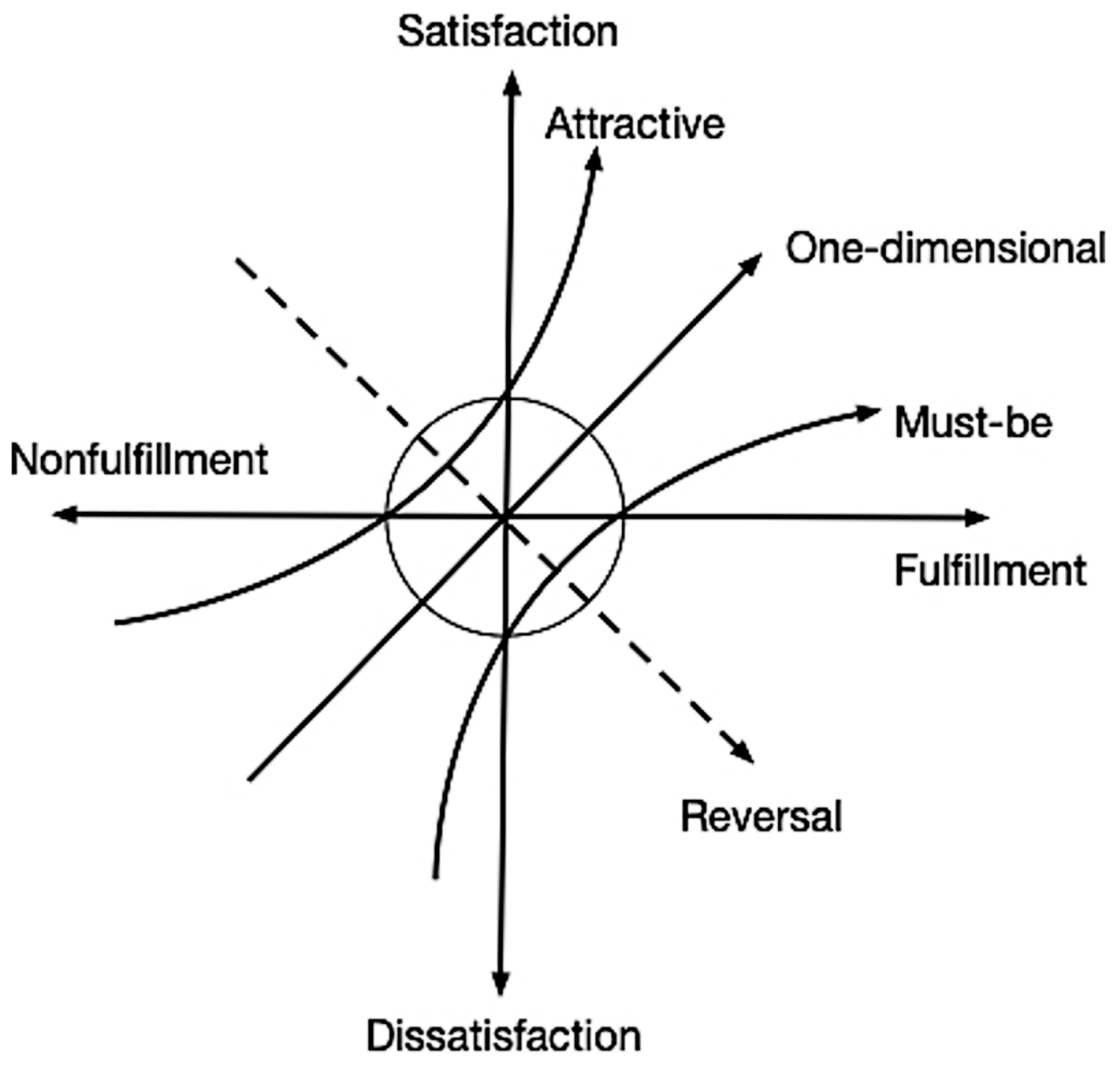
Kano quadrant diagram.

*Reverse attributes*: These are features that may be perceived negatively by some users. Their presence can lead to dissatisfaction, whereas their absence may be evaluated positively (Figure 1).

### Survey method

The questionnaire consisted of two sections. The first section collected respondents’ basic demographic and usage information, including gender, educational background, duration of activity, and frequency of park visits. The second section comprised the Kano questionnaire, which included paired positive and negative questions. For example, a positively worded item was: “If the park provides convenient access, how would you feel?” and its corresponding negative item was: “If the park does not provide convenient access, how would you feel?” Each item offered five response options: I like it that way, It must be that way, I am neutral, I can tolerate it that way, and I dislike it that way. In total, the questionnaire contained 25 paired questions corresponding to the service demand scale.

This study employed a purposive sampling strategy, intentionally selecting sports parks that are well-established and possess high public visibility: Jinan Sports Park, Rizhao Olympic Sports Park, and Weihai Century Sports Park. The selection of these three parks, all at a comparable stage of development, was intended to provide a relatively ideal setting with a sufficient sample size to clearly identify the public sports service demands of older adults in Shandong Province using the Kano model. This methodological choice was critical: if parks lack basic functions (e.g., safety, sanitation), user demands tend to cluster exclusively around ‘must-be’ attributes. This concentration would obscure the identification of more nuanced ‘one-dimensional’ (performance) and ‘attractive’ attributes. Consequently, selecting mature parks allowed for a deeper investigation into the factors that genuinely enhance satisfaction after fundamental needs have already been met.

To address potential challenges, such as varying educational backgrounds and possible reluctance among older adults, investigators received systematic training prior to the survey. This training covered the study’s objectives, questionnaire content, and procedural protocols, ensuring investigators were thoroughly familiar with the instrument and possessed proficiency in local dialects. During the data collection phase, investigators conducted one-on-one, structured interviews. The questionnaire items were administered orally by the investigator, and the respondents’ answers were recorded. All questionnaires were retrieved on-site immediately upon completion. Based on preliminary field investigations, the survey was conducted between May and July 2025. Participants (aged 60 and above) were randomly sampled within the sports parks during peak usage hours, encompassing both mornings and evenings on weekdays and weekends. A total of 900 questionnaires were administered (300 per park). After excluding incomplete or invalid responses, 838 valid questionnaires were obtained, yielding an effective response rate of 93.1%.

Drawing on previous studies (30–33), the questionnaire was structured around six dimensions: safety of park activities, convenience of park use, environmental comfort, facility diversity, openness and sharing, and suitability for social interaction, covering a total of 25 service attributes. The second part included 25 pairs of questions, each consisting of a positive (functional) and a negative (dysfunctional) form. For example: *“1A. If surveillance sites are integrated into community services, how would you feel?”* and *“1B. If such sites are not available, how would you feel?”* For each question, respondents selected from five possible answers: *I like it that way*, *It must be that way*, *I am neutral*, *I can tolerate it that way*, or *I dislike it that way*. Each response pattern corresponded to one Kano attribute: “M” for must-be, “O” for one-dimensional, “A” for attractive, “I” for indifferent, “R” for reverse, and “Q” for questionable results, as summarized in Table 1.

**Table 1:** Distribution of service quality attributes based on the Kano model.

Functional form	Dysfunctional form				
	I like it that way	It must be that way	I am neutral	I can live with it	I dislike it that way
I like it that way	Q	A	A	A	O
It must be that way	R	I	I	I	M
I am neutral	R	I	I	I	M
I can live with it	R	I	I	I	M
I dislike it that way	R	R	R	R	Q

This study employed Cronbach’s *α* coefficient to test the reliability of the questionnaire, a measure first introduced by L. J. Cronbach (34). The coefficient ranges from 0 to 1, with values closer to 1 indicating higher reliability. The survey data were imported into SPSS 26.0 for reliability analysis, and Cronbach’s *α* values were calculated separately for the positive and negative items of older adults’ public sports service demands. The *α* coefficients for the positive items across all dimensions ranged from 0.909 to 0.944, with an overall value of 0.930. For the negative items, the coefficients ranged from 0.833to 0.908, with an overall value of 0.868. In general, a Cronbach’s *α* greater than 0.6 indicates acceptable internal consistency; values between 0.7 and 0.8 suggest good reliability, while values between 0.8 and 0.9 reflect very high reliability. Furthermore, the Kano questionnaire yielded a Kaiser-Meyer-Olkin (KMO) value of 0.946, and Bartlett’s test of sphericity was significant (*p* < 0.001), confirming that the data were suitable for factor analysis. These results demonstrate that the scale has strong reliability and internal consistency, and the measurement outcomes are robust and trustworthy (Tables 2, 3).

**Table 2:** Kano questionnaire dimensions and service items.

Dimension	Number	Service attribute
A. Activity safety	A1	Site protection
	A2	Public security
	A3	Lighting system
	A4	Emergency facilities
B. Accessibility	B1	Convenient access
	B2	Public service facilities
	B3	Barrier-free facilities
C. Environmental comfort	C1	Environmental quality
	C2	Spatial comfort
	C3	Pavement quality
	C4	Maintenance management
D. Facility diversity	D1	Sports facilities
	D2	Recreational fitness facilities
	D3	Resting facilities
	D4	Greening facilities
E. Openness and sharing	E1	Multi-sharing
	E2	All-day access
	E3	Social adaptability
	E4	Sports activity information
	E5	Health education
F. Activity provision	F1	Professional fitness guidance
	F2	Recreational sports activities
	F3	Competitive sports activities
	F4	Sports skills training
	F5	Health monitoring activities

**Table 3:** Functional and dysfunctional values across dimensions.

Dimension	Functional question	Dysfunctional question	Num
A. Activity safety	0.909	0.854	4
B. Accessibility	0.936	0.855	3
C. Environmental comfort	0.938	0.833	4
D. Facility diversity	0.928	0.854	4
E. Openness and sharing	0.926	0.901	5
F. Activity provision	0.944	0.908	5
Overall	0.930	0.868	25

## Discussion

Using the Kano model, this study investigated the needs of older adults aged 60 and above in Jinan, Rizhao, and Weihai, Shandong Province, across 25 service items related to sports parks. Among the respondents, 513 were male (61.22%) and 325 were female (38.78%), indicating that the proportion of male users was significantly higher than that of female users. In terms of age distribution, the majority of respondents were younger-old adults. The largest group was aged 60–65, comprising 529 individuals, followed by the 66–70 age group with 235 individuals. Together, these two groups accounted for more than 90% of the sample. In contrast, the proportions of older adults aged 71–75, 76–80, and over 80 were 5.25, 2.74, and 0.84%, respectively, showing a marked decline with increasing age. Educational attainment was concentrated primarily at the middle school level or below. Regarding mobility patterns, the vast majority of older adults reported travel times of less than 20 min, while only 28.04% had travel times exceeding 20 min. This finding suggests that short-distance travel is the predominant mode. Walking was by far the dominant form of transport, chosen by 611 respondents, representing 72.91% of the sample (Table 4).

**Table 4:** Demographic characteristics.

Characteristics	Observation (*N*)	Percentage (%)
Gender		
Male	513	61.22%
Female	325	38.78%
Age group (years)		
60–65	529	63.13%
66–70	235	28.04%
71–75	44	5.25%
76–80	23	2.74%
80 and above	7	0.84%
Education level		
Secondary school or below	594	70.88%
College or bachelor degree	193	23.03%
Master degree or higher	51	6.09%
Current living condition		
Living alone	124	14.79%
With spouse	478	57.04%
With children	227	27.08%
Other	9	1.09%
Distance		
< 20 min	603	71.96%
≥ 20 min	235	28.04%
Transport options		
Walking	611	72.91%
Non-motorized transport	227	27.09%

Analysis of the Kano questionnaire produced the following results. Indifferent attributes accounted for the largest proportion (36.0%), indicating that for more than one-third of the service items, their presence or absence had little impact on older users’ satisfaction. For example, B1 (convenient access) and E2 (all-day opening) were classified into this category, suggesting either that these functions had already met user expectations in the sampled parks or that they were not central concerns for this demographic group. Attractive and must-be attributes represented comparable proportions, together forming the critical dimensions influencing user satisfaction. Attractive attributes (e.g., E5 health education) function as potential levers for enhancing satisfaction, whereas must-be attributes (e.g., B3 barrier-free facilities) serve as essential conditions to prevent dissatisfaction. One-dimensional attributes accounted for 16.0% of the total, with features such as A1 site protection showing a linear relationship between performance and satisfaction. Specifically, B2, D1, E1, F1, and F4 were classified as must-be qualities; A1, C2, D2, and F2 as one-dimensional qualities; A3, C1, C4, D4, E3, E5, and F5 as attractive qualities; and A2, A4, B1, B3, C3, D3, E2, E4, and F3 as indifferent qualities. Overall, the demand structure of older adults in community sports parks exhibited a clear hierarchy, with different types of attributes exerting varying degrees of influence on satisfaction (Table 5).

**Table 5:** Service quality statistics.

Num	Service attribute	A	O	M	I	Attribute
A. Activity safety						
A1	Site protection	212	257	225	144	O
A2	Public security	208	141	201	288	I
A3	Lighting system	267	239	198	134	A
A4	Emergency facilities	200	164	210	264	I
B. Accessibility						
B1	Convenient access	231	202	173	232	I
B2	Public service facilities	217	197	340	84	M
B3	Barrier-free facilities	222	152	157	307	I
C. Environmental comfort						
C1	Environmental quality	277	185	155	221	A
C2	Spatial comfort	213	281	213	131	O
C3	Pavement quality	221	185	150	282	I
C4	Maintenance management	309	206	172	151	A
D. Facility diversity						
D1	Sports facilities	131	243	260	204	M
D2	Recreational fitness facilities	203	296	205	134	O
D3	Resting facilities	221	211	136	270	I
D4	Greening facilities	258	209	172	199	A
E. Openness and sharing						
E1	Multi-sharing	229	216	260	133	M
E2	All-day access	253	173	126	286	I
E3	Social adaptability	283	243	170	142	A
E4	Sports activity information	211	202	163	262	I
E5	Health education	258	209	172	199	A
F. Activity provision						
F1	Professional fitness guidance	175	240	259	164	M
F2	Recreational sports activities	203	302	205	128	O
F3	Competitive sports activities	221	212	133	272	I
F4	Sports skills training	132	238	267	201	M
F5	Health monitoring activities	257	197	176	208	A

Must-be attributes accounted for 20% of the total. The defining feature of these attributes is that their absence generates strong dissatisfaction. For instance, items such as public service facilities (B2), sports facilities (D1), multi-sharing (E1), professional fitness guidance (F1) and sports skills training (F4) fall into this category. These services represent the minimum requirements for older adults when using sports parks, and any deficiencies directly undermine their user experience. Therefore, such services must be guaranteed to avoid negative impacts on older adults users. For must-be demands, accessibility, safety, and functional adequacy of facilities are indispensable, as improvements in these areas directly affect the basic quality of life for older adults.

Attractive attributes comprised 28%, a relatively high proportion in this study. Service items such as lighting systems (A3), environmental quality (C1), maintenance management (C4), greening facilities (D4), social adaptability (E3), health education (E5) and health monitoring activities (F5) were categorized as attractive attributes. The defining feature of attractive demands is that their presence significantly enhances satisfaction, whereas their absence generally does not provoke dissatisfaction. Enhancing such services can rapidly improve overall well-being among older adults and foster positive word-of-mouth. In particular, services related to social engagement and personalized guidance can substantially increase older adults’ reliance on and participation in public spaces, thereby reinforcing their sense of social belonging.

One-dimensional attributes accounted for 16%. These are services that older adults expect to be available. Although their absence does not cause severe dissatisfaction, their presence greatly improves satisfaction. Items such as site protection (A1), spatial comfort (C2), recreational fitness facilities (D2) and recreational sports activities (F2) fall into this category. These demands are relatively flexible and can effectively enhance satisfaction levels. Given the dynamic nature of social and individual needs, one-dimensional attributes may evolve into attractive or even must-be demands over time. Thus, service providers should continuously monitor such changes and make timely adjustments to maintain high satisfaction levels among older adults.

In this survey, indifferent attributes accounted for the largest share (36%). Specifically, the presence or absence of these attributes had minimal impact on satisfaction. Items such as public security (A2), emergency facilities (A4), convenient access (B1), barrier-free facilities (B3), pavement quality (C3), resting facilities (D3), all-day access (E2), sports activity information (E4), competitive sports activities (F3) were categorized as indifferent. Older adults responded rather neutrally to these attributes, indicating that their demand for such functions is not urgent. Consequently, resource allocation for these services can be moderately reduced, allowing providers to concentrate on improving service quality in other, more critical categories.

The Better value measures the feature’s ability to generate satisfaction, while the Worse value estimates the potential dissatisfaction caused by its omission (35). The calculation formulas for the better and worse values are as follows:


Better=(A+O)/(A+M+O+I)

$$\text{Worse}=({\left(-1\right)}^{*}(O+M))/(A+M+O+I)$$

From a dimensional perspective, this study involved six dimensions: A. Activity safety, B. Accessibility, C. Environmental Comfort, D. Facility Diversity, E. Openness and Sharing, and F. Activity Provision. The mean better and worse values of each dimension reveal their respective roles in enhancing satisfaction and preventing dissatisfaction (Tables 6, 7).

**Table 6:** Statistical results of better–worse values for individual services.

Num	Service attribute	Better	Worse
Dimension A. Activity safety			
A1	Site protection	0.5597	0.5752
A2	Public security	0.4165	0.4081
A3	Lighting system	0.6038	0.5215
A4	Emergency facilities	0.4463	0.4582
Dimension B. Accessibility			
B1	Convenient access	0.5167	0.4475
B2	Public service facilities	0.4940	0.6408
B3	Barrier-free facilities	0.4463	0.3687
Dimension C. Environmental comfort			
C1	Environmental quality	0.5501	0.4057
C2	Spatial comfort	0.5883	0.5895
C3	Pavement quality	0.4833	0.3998
C4	Maintenance management	0.6134	0.4511
Dimension D. Facility diversity			
D1	Sports facilities	0.4451	0.6002
D2	Recreational fitness facilities	0.5943	0.5979
D3	Resting facilities	0.5143	0.4141
D4	Greening facilities	0.5561	0.4547
Dimension E. Openness and sharing			
E1	Multi-sharing	0.5298	0.5680
E2	All-day access	0.5072	0.3568
E3	Social adaptability	0.6265	0.4928
E4	Sports activity information	0.4916	0.4356
E5	Health education	0.3079	0.4547
Dimension F. Activity provision			
F1	Professional fitness guidance	0.4952	0.5955
F2	Recreational sports activities	0.6026	0.6050
F3	Competitive sports activities	0.5167	0.4117
F4	Sports skills training	0.4415	0.6026
F5	Health monitoring activities	0.5418	0.4451

**Table 7:** Statistical results of better–worse values across dimensions.

Dimension	Better	Worse
A. Activity safety	0.5066	0.4908
B. Accessibility	0.4857	0.4857
C. Environmental comfort	0.5588	0.4615
D. Facility diversity	0.5275	0.5167
E. Openness and sharing	0.4926	0.4616
F. Activity provision	0.5196	0.5320
Overall	0.5151	0.4914

The worse values of Facility Diversity (Dimension D) and Activity Provision (Dimension F) were relatively high, indicating that the absence of basic functions in these two dimensions would directly undermine older adults’ evaluations of sports parks. Therefore, these dimensions should be prioritized in infrastructure development to ensure that older adults users can access and utilize community sports parks conveniently and without barriers.

For Environmental comfort (Dimension C), the better value was 0.5363, suggesting that this dimension has a significant effect on increasing satisfaction. In particular, providing a comfortable environment, appropriate spatial layouts, and quality landscape design can substantially enhance older adults’ sense of reliance on and satisfaction with sports parks.

**Figure 2:**
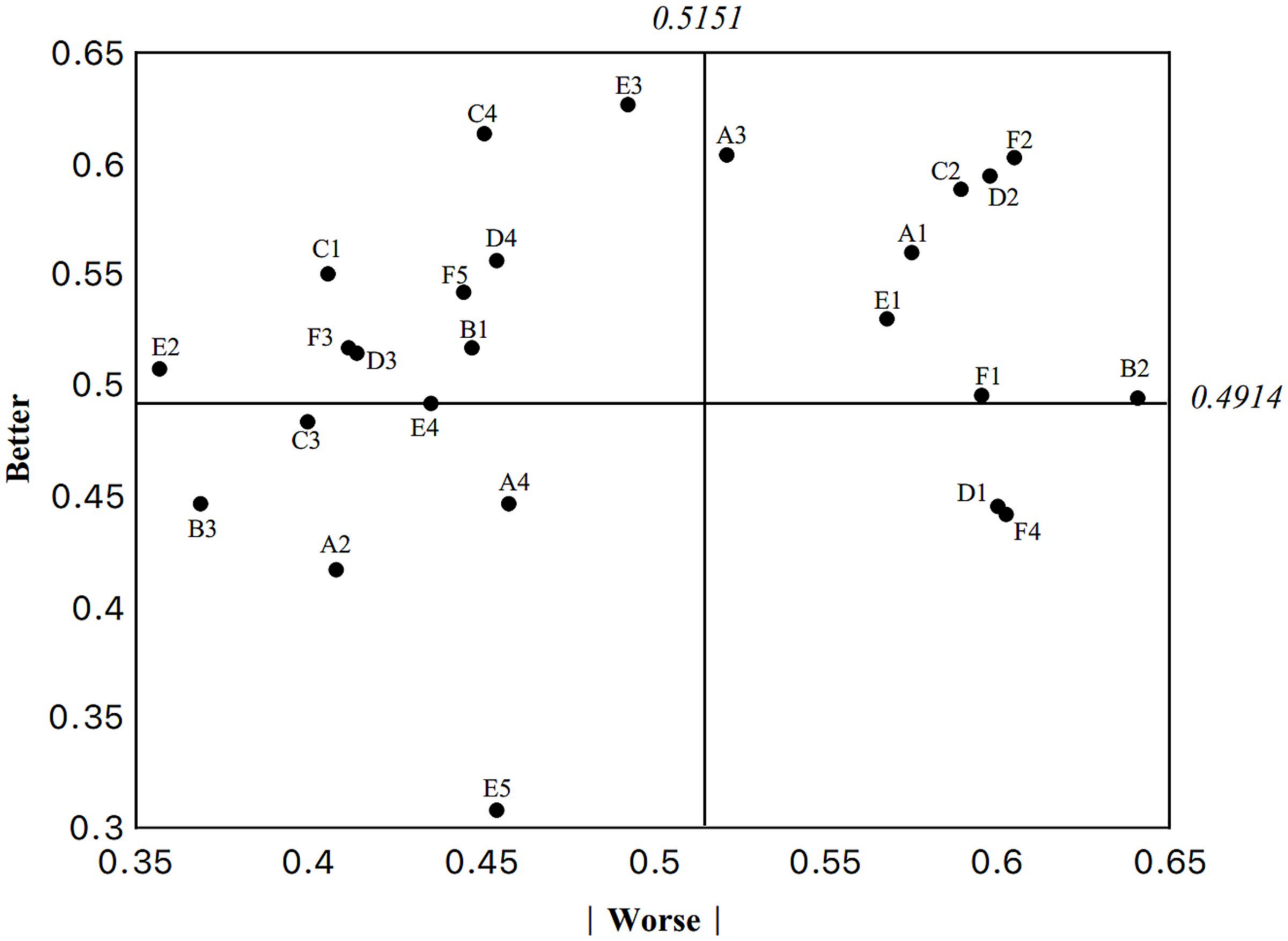
Better–worse coefficient diagram.

The worse value of Activity provision (Dimension F) was 0.5320, indicating that insufficient supply of activities in this dimension would quickly lead to dissatisfaction. Thus, ensuring diversity and timely renewal of activities is critical for sustaining user engagement in this dimension (Figure 2).

To translate the study’s findings into actionable strategies, this paper presents a prioritized action checklist for park managers and urban planners:

Priority 1: Securing “Must-be Attributes” (Preventing Dissatisfaction)

Managers must primarily allocate resources to guarantee the full functionality of all “Must-be attributes,” specifically public service facilities (B2), sports facilities (D1), multi-sharing (E1), professional fitness guidance (F1), and sports skills training (F4). Any negligence in these fundamental functions will precipitate strong user dissatisfaction and negate investments made in other areas.

Priority 2: Optimizing “One-dimensional Attributes” (Enhancing Satisfaction)

Once essential needs are met, proportional investments should be made in “One-dimensional attributes,” as they exhibit a linear positive correlation with satisfaction. Key attributes include site protection (A1), spatial comfort (C2), recreational fitness facilities (D2), and recreational sports activities (F2). Improvements in these facilities—such as installing more comfortable seating or offering a wider variety of leisure activities—will directly yield steady increases in user satisfaction.

Priority 3: Leveraging “Attractive Attributes” (Creating Excellence)

“Attractive attributes” are critical for achieving service differentiation and generating positive word-of-mouth. Where budgets permit, investing in lighting systems (A3), environmental quality (C1), maintenance management (C4), greening facilities (D4), social adaptability (E3), health education (E5), and health monitoring activities (F5) can significantly enhance older users’ sense of surprise and belonging.

Re-evaluating “Indifferent Attributes” Regarding attributes classified as “Indifferent attribute”—namely public security (A2), emergency facilities (A4), convenient access (B1), barrier-free facilities (B3), pavement quality (C3), resting facilities (D3), all-day access (E2), sports activity information (E4), and competitive sports activities (F3)—managers should not dismiss them as unimportant. As noted in the “Limitations” section, this classification likely indicates that these facilities have already met the baseline standards to which users are accustomed in the high-quality parks studied. Conversely, for newly constructed parks or those with inferior infrastructure, some of these attributes may in fact constitute “Must-be attributes.”

### Suggestions

#### Ensuring safety in park use

The results indicate that convenient access, barrier-free facilities, and adequate lighting are fundamental requirements for older adults using community sports parks. Poorly designed height differences within the park may cause inconvenience to older adults users. To ensure safety and accessibility, barrier-free facilities should be installed at main entrances and frequently used activity areas. Additionally, sufficient and well-distributed lighting systems should be provided to guarantee safety during nighttime activities. Park authorities should also strengthen the maintenance of easily worn facilities, such as fitness equipment and walking paths, while promptly addressing vandalism of public facilities through appropriate guidance and intervention, thereby promoting sustainable service for community groups.

#### Improving the quality of activity spaces

The findings reveal that older adults place high value on leisure and fitness facilities as well as environmental comfort. Walking paths, in particular, effectively meet their needs for strolling and social interaction. In practical design, continuity and safety of routes should be emphasized, minimizing interference from external traffic or unsafe conditions. To improve spatial comfort, shade structures such as pavilions and trellises should be added in areas lacking natural tree cover to reduce sun exposure during summer. Given the diversity of activity preferences, quiet spaces for static activities such as chess, tai chi, and social gatherings should be separated from dynamic activity zones such as ball games and square dancing to minimize mutual disturbance between groups.

#### Enhancing park facilities

Following retirement, many older adults face feelings of loneliness and social disconnection. Engaging in physical exercise and integrating into community life can improve their sense of happiness. Thus, the provision of appropriate facilities plays a vital role in supporting diverse activities. Resting facilities are essential, as declining physical capacity increases the need for seating. Parks should be equipped with adequate and comfortable resting areas, such as benches with backrests, particularly in locations frequently used by older adults. For those engaging in sports like table tennis and badminton, safety can be ensured by providing boundary fences and slip-resistant flooring. In addition, many older adults care for grandchildren on a daily basis; hence, incorporating playground facilities such as climbing frames, swings, and slides can enhance intergenerational interaction and foster a shared atmosphere. Vegetation planning should also emphasize a balanced level of greenery and diversity. Both excessive and insufficient vegetation may hinder activities, whereas a green coverage ratio of 40–50% has been shown to prolong users’ stay in the park.

#### Promoting multi-stakeholder park management

In October 2021, seven ministries jointly issued the Guidelines on Promoting the Construction of Sports Parks, highlighting convenience and public accessibility as fundamental principles of community sports parks. The policy also encouraged third-party enterprises to participate in park construction and management. Under this model, governments delegate responsibilities for site selection, infrastructure development, and daily operations to third-party organizations (36). Such entities bring strong expertise, extensive experience, and relatively lower operational costs, which not only alleviate government workload but also strengthen the delivery of community sports services. Accordingly, the development of age-friendly community sports parks should actively integrate third-party organizations, including enterprises, industry associations, community sports clubs, local associations, and volunteer teams, to establish diversified management models. Allowing micro-profit operations can further improve efficiency in operation and management, ultimately fostering an age-friendly and livable environment for older adults. Finally, the results suggest that, compared to other environmental factors, site protection, public security, and multi-stakeholder management exert relatively little influence on older adults’ satisfaction. This implies that older adults users place greater emphasis on the physical infrastructure and the overall comfort of the spatial environment. As community sports parks serve multiple age groups, they should balance the needs of older adults with those of other users, adhering to principles of intergenerational sharing and integration. By doing so, parks can create a vibrant atmosphere that sustains their appeal and strengthens community vitality.

### Limitations and future research

First, sampling bias is a primary limitation. This study employed purposive sampling, selecting three sports parks with well-developed infrastructure and mature management. This selection led to an ‘indifferent’ classification for service attributes (specifically B3 “Barrier-free facilities “and B1 “Convenient access “), as these basic needs were likely already well-satisfied in the sampled parks. Consequently, the findings cannot be generalized to parks with poorer infrastructure or newly built ones, where accessibility would very likely remain a must-be attribute. Second, the demographic characteristics of this study are significantly skewed due to the random sampling of older adult individuals within the sports parks. Future research should adopt a stratified sampling strategy to ensure a more accurate assessment of these underrepresented groups, whose mobility, safety needs, and social preferences may differ significantly. Third, there are limitations related to measurement and statistical analysis. The high Cronbach’s *α* coefficients suggest potential item redundancy within the dimensions, indicating a need for questionnaire refinement in the future.

## Data Availability

The original contributions presented in the study are included in the article/Supplementary material, further inquiries can be directed to the corresponding author.
