# Supplementary material for: Older adults’ demands for sports parks: a Kano model study based on sports parks in Shandong Province of China
Source: Front Public Health. 2025 Dec 29;13:1703037. doi: 10.3389/fpubh.2025.1703037 (PMC12793901; doi:10.3389/fpubh.2025.1703037)
Supplement: Supplementary file 2 [file Table_2.DOCX]

Questionnaire Validity Test

| Item | Factor Loading Coefficient | | | | | Common Factor Variance |
| --- | --- | --- | --- | --- | --- | --- |
|  | **Factor 1** | **Factor 2** | **Factor 3** | **Factor 4** | **Factor 5** |  |
| A1（Positive） | 0.873 | -0.112 | 0.161 | -0.336 | -0.076 | 0.919 |
| A1（Reverse） | 0.160 | 0.880 | -0.022 | -0.159 | 0.130 | 0.843 |
| A2（Positive） | 0.865 | 0.003 | 0.060 | 0.221 | -0.110 | 0.813 |
| A2（Reverse） | -0.129 | 0.888 | -0.183 | 0.117 | 0.016 | 0.853 |
| A3（Positive） | 0.847 | -0.158 | 0.013 | -0.308 | -0.203 | 0.878 |
| A3（Reverse） | 0.237 | 0.670 | 0.604 | 0.068 | -0.200 | 0.914 |
| A4（Positive） | 0.883 | 0.135 | 0.068 | 0.038 | -0.020 | 0.804 |
| A4（Reverse） | -0.068 | 0.825 | -0.302 | 0.178 | 0.231 | 0.862 |
| B1（Positive） | 0.947 | 0.008 | -0.013 | 0.003 | 0.077 | 0.903 |
| B1（Reverse） | -0.105 | 0.890 | -0.022 | 0.140 | 0.126 | 0.839 |
| B2（Positive） | 0.945 | 0.027 | 0.022 | 0.097 | 0.064 | 0.907 |
| B2（Reverse） | 0.238 | 0.827 | 0.022 | -0.096 | -0.181 | 0.783 |
| B3（Positive） | 0.876 | 0.005 | 0.197 | 0.134 | 0.039 | 0.826 |
| B3（Reverse） | -0.415 | 0.800 | -0.015 | 0.241 | -0.035 | 0.871 |
| C1（Positive） | 0.931 | -0.061 | 0.002 | -0.117 | -0.004 | 0.884 |
| C1（Reverse） | 0.022 | 0.736 | 0.288 | 0.230 | 0.262 | 0.746 |
| C2（Positive） | 0.898 | -0.141 | -0.074 | -0.215 | -0.111 | 0.890 |
| C2（Reverse） | 0.161 | 0.881 | 0.133 | -0.121 | -0.256 | 0.899 |
| C3（Positive） | 0.901 | -0.136 | 0.075 | 0.154 | 0.016 | 0.861 |
| C3（Reverse） | -0.238 | 0.806 | -0.069 | 0.388 | -0.029 | 0.862 |
| C4（Positive） | 0.853 | -0.157 | -0.023 | -0.319 | -0.182 | 0.888 |
| C4（Reverse） | 0.263 | 0.586 | 0.489 | -0.085 | 0.176 | 0.690 |
| D1（Positive） | 0.896 | 0.161 | 0.138 | 0.113 | 0.069 | 0.866 |
| D1（Reverse） | -0.240 | 0.725 | -0.027 | -0.069 | 0.526 | 0.865 |
| D2（Positive） | 0.893 | -0.140 | -0.046 | -0.248 | -0.128 | 0.897 |
| D2（Reverse） | 0.078 | 0.891 | 0.146 | -0.193 | -0.165 | 0.887 |
| D3（Positive） | 0.887 | -0.119 | -0.040 | 0.056 | 0.040 | 0.807 |
| D3（Reverse） | -0.431 | 0.810 | 0.085 | 0.142 | 0.078 | 0.876 |
| D4（Positive） | 0.899 | -0.043 | 0.019 | -0.176 | -0.066 | 0.847 |
| D4（Reverse） | 0.120 | 0.745 | 0.494 | 0.224 | -0.072 | 0.868 |
| E1（Positive） | 0.921 | -0.012 | 0.008 | -0.066 | -0.019 | 0.854 |
| E1（Reverse） | 0.116 | 0.859 | 0.302 | -0.111 | -0.119 | 0.869 |
| E2（Positive） | 0.930 | 0.044 | 0.052 | -0.007 | 0.010 | 0.869 |
| E2（Reverse） | -0.058 | 0.673 | 0.360 | 0.512 | -0.194 | 0.886 |
| E3（Positive） | 0.842 | -0.195 | -0.029 | -0.319 | -0.167 | 0.878 |
| E3（Reverse） | 0.214 | 0.755 | 0.478 | -0.075 | 0.061 | 0.854 |
| E4（Positive） | 0.908 | 0.121 | 0.069 | 0.074 | 0.101 | 0.860 |
| E4（Reverse） | -0.238 | 0.864 | -0.168 | 0.226 | 0.020 | 0.883 |
| E5（Positive） | 0.701 | 0.401 | 0.063 | 0.264 | -0.092 | 0.735 |
| E5（Reverse） | -0.001 | 0.760 | 0.373 | 0.134 | 0.291 | 0.819 |
| F1（Positive） | 0.928 | 0.046 | 0.080 | 0.042 | 0.072 | 0.876 |
| F1（Reverse） | -0.028 | 0.844 | -0.032 | -0.168 | 0.259 | 0.810 |
| F2（Positive） | 0.870 | -0.155 | 0.023 | -0.290 | -0.146 | 0.887 |
| F2（Reverse） | 0.104 | 0.893 | 0.086 | -0.171 | -0.166 | 0.872 |
| F3（Positive） | 0.915 | -0.097 | -0.023 | 0.090 | 0.021 | 0.856 |
| F3（Reverse） | -0.226 | 0.853 | -0.117 | 0.297 | -0.084 | 0.888 |
| F4（Positive） | 0.881 | 0.148 | 0.147 | 0.117 | 0.077 | 0.838 |
| F4（Reverse） | -0.235 | 0.730 | -0.022 | -0.075 | 0.516 | 0.861 |
| F5（Positive） | 0.876 | -0.030 | 0.014 | -0.145 | -0.184 | 0.823 |
| F5（Reverse） | -0.032 | 0.803 | 0.304 | 0.119 | 0.258 | 0.820 |
| **Eigenvalue (Initial)** | 21.312 | 16.958 | 1.659 | 1.551 | 1.204 | - |
| **% of Variance (Initial)** | 42.623% | 33.916% | 3.319% | 3.103% | 2.408% | - |
| **Cumulative % (Initial)** | 42.623% | 76.539% | 79.858% | 82.961% | 85.368% | - |
| **Eigenvalue (Rotated)** | 20.705 | 16.609 | 1.991 | 1.905 | 1.474 | - |
| **% of Variance (Rotated)** | 41.410% | 33.218% | 3.982% | 3.810% | 2.948% | - |
| **Cumulative % (Rotated)** | 41.410% | 74.628% | 78.610% | 82.421% | 85.368% | - |
| **KMO Value** | 0.946 | | | | | - |
| **Bartlett's Test Value** | 81015.305 | | | | | - |
| *df* | 1225 | | | | | - |
| **p-value** | 0.000 | | | | | - |
